# Autoimmune arthritis caused by altered thymic T-cell selection due to a mutation of the ZAP-70 gene

**DOI:** 10.1186/ar3565

**Published:** 2012-02-09

**Authors:** Yoshinaga Ito, Shimon Sakaguchi

**Affiliations:** 1 Department of Experimental Pathology, Institute for Frontier Medical Sciences, Kyoto University, Kyoto, Japan; 2 Laboratory of Experimental Immunology, WPI Immunology Frontier Research Center, Osaka University, Suita, Japan

## 

SKG mouse is a murine model of autoimmune arthritis. A spontaneous point mutation of the gene encoding an SH2 domain of the ζ-associated protein of 70 kDa gene (ZAP-70), a key signal transduction molecule in T cells, causes chronic autoimmune arthritis in SKG mice that resembles human RA in many aspects. Altered signal transduction from T-cell antigen receptor through the aberrant ZAP-70 changes the thresholds of T cells to thymic selection, leading to the positive selection of otherwise negatively selected autoimmune T cells.

Based on the finding that the skg-mutation of ZAP-70 causes autoimmune arthritis, we then examined how attenuated TCR signaling affects the spectrum of autoimmune diseases. In a set of mice with the mutation, the amount of ZAP-70 protein as well as its tyrosine phosphorylation upon TCR stimulation decreased from +/+, *skg*/+, *skg*/*skg*, to *skg*/− mice in a stepwise manner. The reduction resulted in graded alterations of thymic positive and negative selection of self-reactive T cells and Foxp3^+ ^natural regulatory T cells (Tregs) and their respective functions. Consequently, *skg*/− mice spontaneously developed autoimmune arthritis even in a microbially clean environment, whereas *skg*/*skg *mice required stimulation through innate immunity for disease manifestation. After Treg depletion, organ-specific autoimmune diseases, especially autoimmune gastritis, predominantly developed in *+/+*, at a lesser incidence in *skg/+*, but not in *skg*/*skg *BALB/c mice, which suffered from other autoimmune diseases, especially autoimmune arthritis. In correlation with this change, gastritis-mediating TCR transgenic T cells were positively selected in +/+, less in *skg/+*, but not in *skg/skg *BALB/c mice. Similarly, on the genetic background of diabetes-prone NOD mice, diabetes spontaneously developed in *+/+*, at a lesser incidence in *skg/+*, but not in *skg/skg *mice, which instead succumbed to arthritis. Thus, the graded attenuation of TCR signaling alters the repertoire and the function of autoimmune T cells and natural Tregs in a progressive manner. It also changes the dependency of disease development on environmental stimuli. These findings collectively provide a model of how genetic anomaly of T cell signaling contributes to the development of autoimmune disease.

